# Supplementary material for: Mitochondrial ubiquitin ligase alleviates Alzheimer’s disease pathology via blocking the toxic amyloid-β oligomer generation
Source: Commun Biol. 2021 Feb 12;4:192. doi: 10.1038/s42003-021-01720-2 (PMC7881000; doi:10.1038/s42003-021-01720-2)

Supplementary information

Mitochondrial ubiquitin ligase alleviates Alzheimer's disease pathology via blocking the toxic amyloid-β oligomer generation

Keisuke Takeda[1#], Aoi Uda[1], Mikihiro Mitsubori[1], Shun Nagashima[1], Hiroko Iwasaki[1], Naoki Ito[1,2], Isshin Shiiba[1,2], Satoshi Ishido[3], Masaaki Matsuoka[4], Ryoko Inatome[1,2], and Shigeru Yanagi[1,2] *

[1] Laboratory of Molecular Biochemistry, School of Life Sciences, Tokyo University of Pharmacy and Life Sciences, Hachioji, Tokyo, 192-0392, Japan

[2] Laboratory of Molecular Biochemistry, Department of Life Science, Faculty of Science, Gakushuin University, 1-5-1 Mejiro, Toshima-ku, Tokyo, 171-8588, Japan

[3] Department of Microbiology, Hyogo College of Medicine, 1-1, Mukogawa-cho, Nishinomiya, 663-8501, Japan

[4] Department of Pharmacology, Tokyo Medical University, 6-1-1 Shinjuku, Shinjuku-ku, Tokyo, 160-8402, Japan

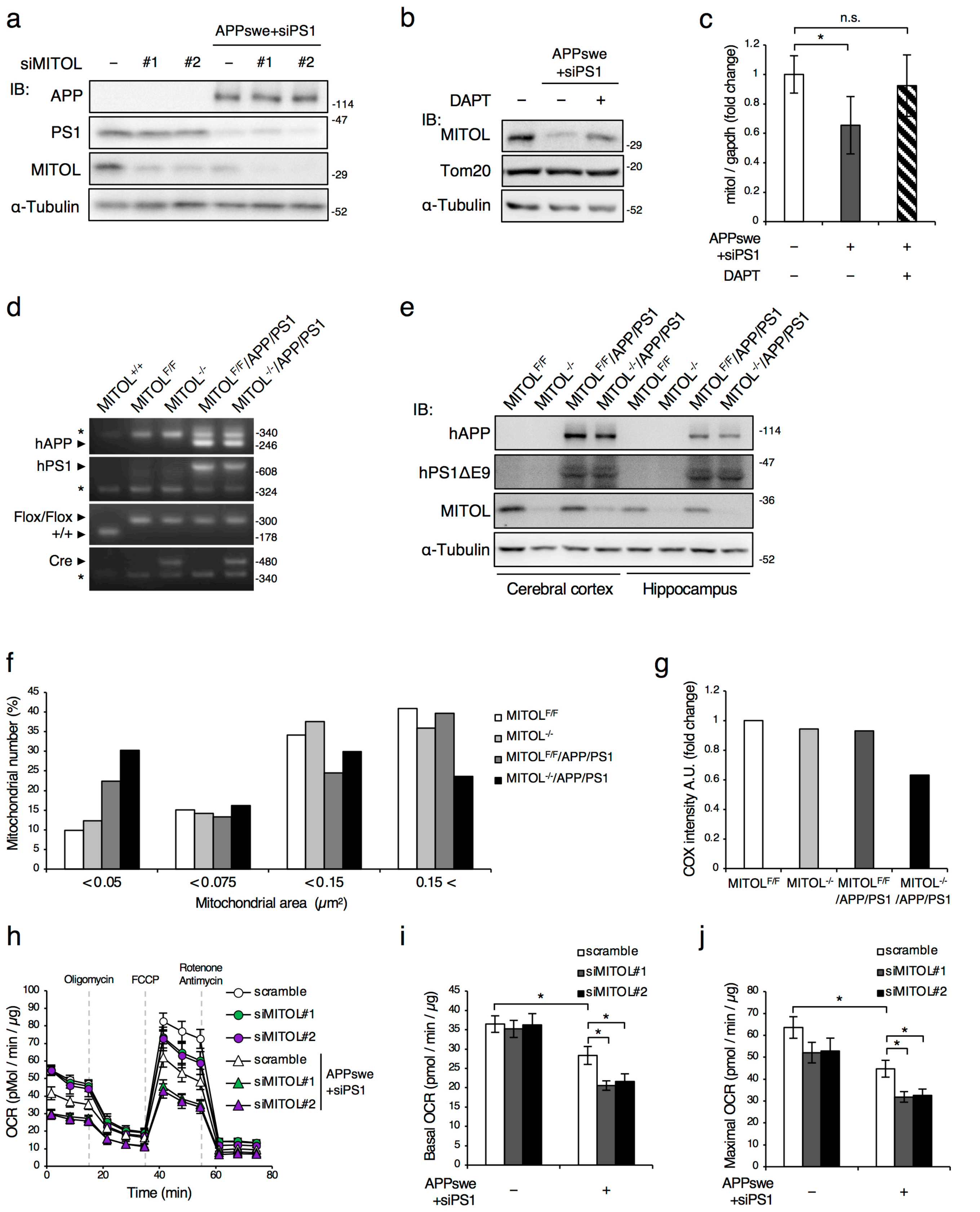

Supplementary Fig. 1: Generation of MITOL-deleted APP/PS1 mice.

a Immunoblot analysis showing the efficiency of indicated siRNAs in SH-SY5Y cells with or without stably expressing APPswe. Cells were transfected with scramble siRNA (scramble), siPS1, or two types of siMITOL (#1, #2) 48 hour before cell lysis. **b**, **c** Aβ production-dependent MITOL repression. SH-SY5Y cells stably expressing APPswe were transfected with siPS1 48 hours before each analysis (**b**, **c**). As control cells, SH-SY5Y without stably expression were transfected with scramble siRNA. These cell lysates were immunoblotted with indicated antibodies (**b**) or analyzed by qRT-PCR (**c**). 30 μM

γ-secretase inhibitor DAPT or DMSO was treated with indicated cells for 15 hours before the analysis. Error bars indicate ± SD (**c**: n=4). *$p<0.05$, n.s.: not significant (Student's t test). (**d**, **e**) Generation of MITOL-deleted APP/PS1 mice. MITOL-deleted mice was generated, as previously reported [19], using Emx1-Cre transgenic mice. APP/PS1 transgenes contain human APPswe and human PS1ΔE9 under PrP promoter. APPswe: APP with Swedish mutation, human PS1ΔE9: PS1 with deletion of exon 9. Genotyping of indicated mice at 1 months of age by genome PCR (**d**). Genomic PCR generated the 246 bp bands for human APP (hAPP), the 608bp bands for human PS1 (hPS1), the 300 bp bands for Floxed allele, the 178bp bands for wild type allele (+/+), the 480 bp bands for Cre recombinase and the 340 bp bands for IL-6, shown as *, as controls. **e** Lysates of cerebral cortex and hippocampus of indicated 3-months old mice were immunoblotted with indicated antibodies. **f** MITOL deletion in APP/PS1 brain disturbed mitochondrial network. The statistical graph indicates the distribution of mitochondrial area based on TEM analysis shown in Fig. 1e (n= over 300 mitochondria in the cell body of neurons of indicated cerebral cortex). **g** MITOL deletion in the APP/PS1 brain impaired mitochondrial bioenergetics. Brains of indicated mice at 15 months of age were subjected to COX staining as shown in Fig. 1i. The upper panels of Fig. 1i were quantified using ImageJ (n=2). **h**-**j** MITOL silencing caused severe mitochondrial dysfunction in the cell model with Aβ etiology. SH-SY5Y cells with or without stably expressing APPswe were transfected with indicated siRNAs 48 hour before measuring oxygen consumption rate (OCR) (**h**). Basal (**i**) and Maximal (**j**) were calculated as described in method. Error bars indicate ± SD (n=6). *$p<0.01$ (one-way ANOVA, Tukey's test).

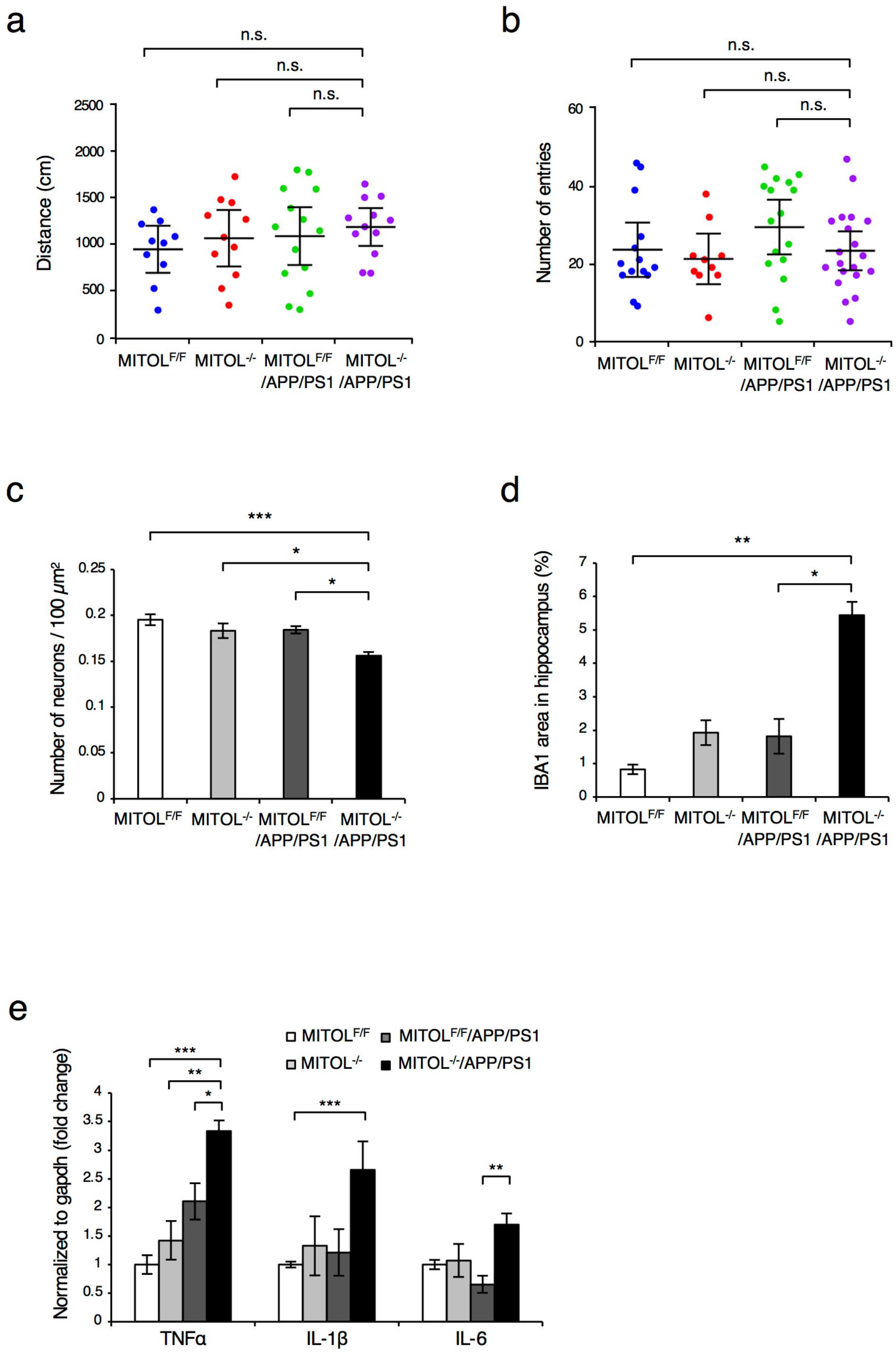

Supplementary Fig. 2. MITOL deletion induces neuronal atrophy and microglial activation in APP/PS1 mice.

a, **b** MITOL deletion did not effect on the activity of mice. Total distance traveled of indicated mice at 15 months of age in the open-field test was quantified using Meander (**a**). The total number of arm entries in Y-maze test for indicated mice at 15 months of age was measured (**b**). Error bars indicate ± SE (**a**: n=9-15, **b**: n=8-21). **c** MITOL deletion enhanced the neuronal atrophy. Brain sections were stained using cresyl violet as shown in Fig. 2d. The number of neurons in the stained sections were counted by visual inspection. Error bars indicate ± SE (n=5-6). ***p<0.001, *p<0.05 (one-way ANOVA, Tukey's test). **d** The quantification of IBA1-positive area. Brain sections of indicated mice at 12 months of age were immunostained with anti-IBA1 antibody as shown in Fig. 2h. The percentage of IBA1-positive area was calculated using ImageJ. Error bars indicate ± SE (**f**: n=4-8). **p<0.01, *p<0.05 (one-way ANOVA, Tukey's test). **e** Enhanced cytokines production in MITOL-deleted APP/PS1 brain. The levels of

pro-inflammatory cytokines TNFα, IL-1β, and IL-6 were evaluated by qRT-PCR in the hippocampus of indicated mice at 12 months of age. Error bars indicate ± SE (**g**: n=4-5, **i**: n=4-6). ***$p<0.001$, **$p<0.01$, *$p<0.05$ (one-way ANOVA, Tukey's test).

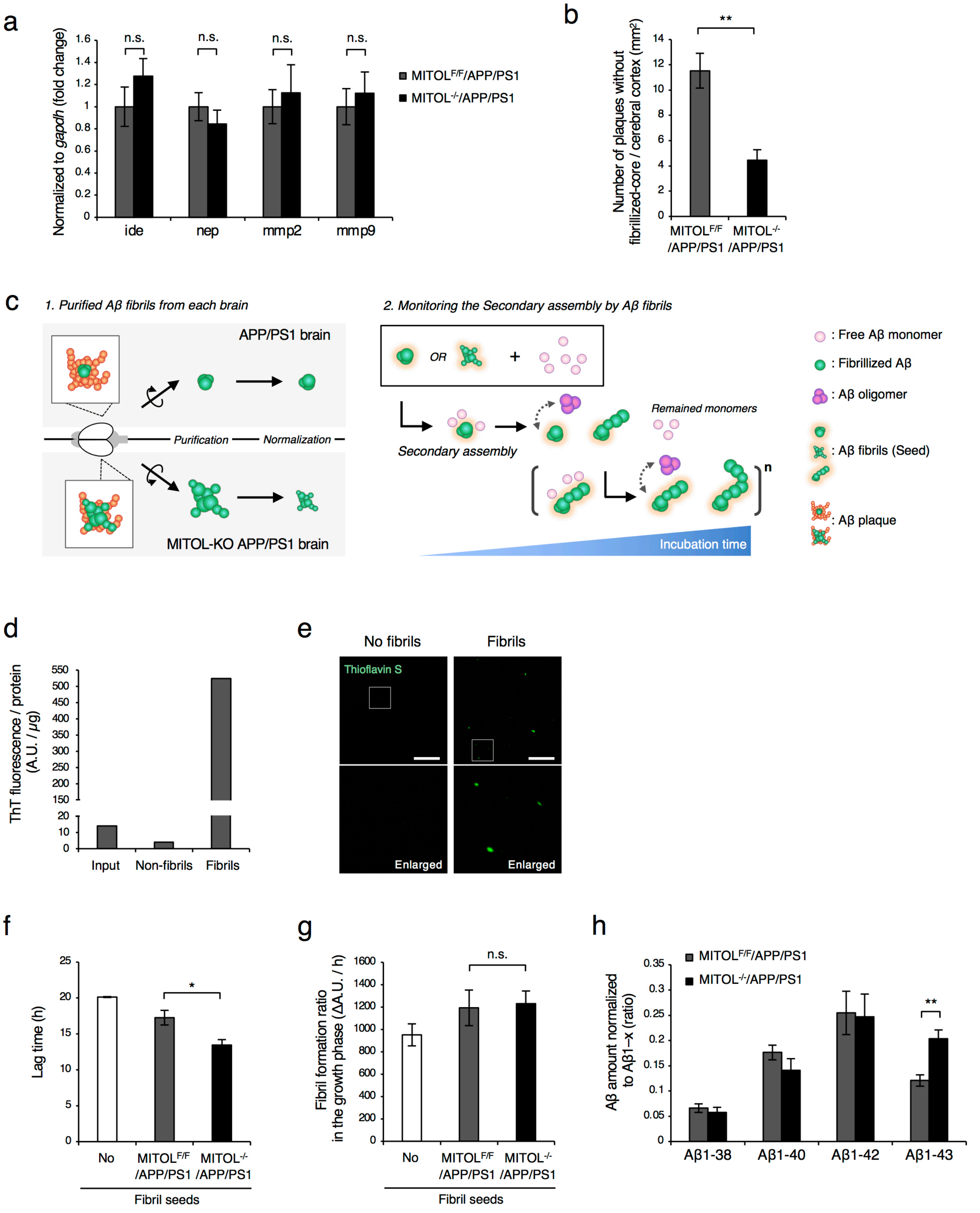

Supplementary Fig. 3. MITOL is dispensable to APP metabolism.

a MITOL deletion unaffected gene expressions of Aβ-degrading enzymes. mRNA was extracted from the hippocampus of indicated mice at 15 months of age, followed by qRT-PCR. Error bars indicate ± SE (n=4-5). n.s.: not significant (Student's t test). **b** MITOL deletion reduced the formation of non-toxic Aβ plaques. The number of non-toxic Aβ plaques, without fibrillized regions, were calculated from the staining in Fig. 3d according to processes as mentioned in method. **c** The working diagram of the sequential experiments including Aβ fibrils isolation and measurement of the seeding activity. **d**, **e**

Purification of Aβ fibrils from the APP/PS1 brain. The fibril seeds of APP/PS1 brain at 15 months of age were isolated as described in the method. The amounts of fibril seeds in equal protein amounts of each fraction were confirmed with ThT using microplate reader (**d**) or ThS using confocal microscopy (**e**). The lower panels show high-magnification images of the boxed regions. Scale bar represents 100 µm. (**f**, **g**) Aβ fibrils from the MITOL-deleted APP/PS1 brain shortened the time up to the formation of new Aβ fibrils but did not affect the aggregation speed. Aβ fibrils in indicated 15-months old mice were isolated and subjected to the assay for monitoring the seeding activity of the isolated Aβ fibrils as shown in Fig. 3i. The time up to the nucleation point and the fibril formation ratio were calculated from ThT-monitored kinetic curves as lag-phase and elongation rate, respectively. Error bars indicate ± SE (n=4). *$p<0.05$, n.s.: not significant (Student's t test). **h** MITOL deletion caused the formation of Aβ43-enriched Aβ plaques. Aβ fibrils, including fibrillized Aβ plaques, in the 15 month-old mice brain were isolated as described in method. The fibrils were subjected to ELISA measurement for specifically detecting each Aβ species. Error bars indicate ± SE (n=4). **$p<0.01$ (Student's t test).

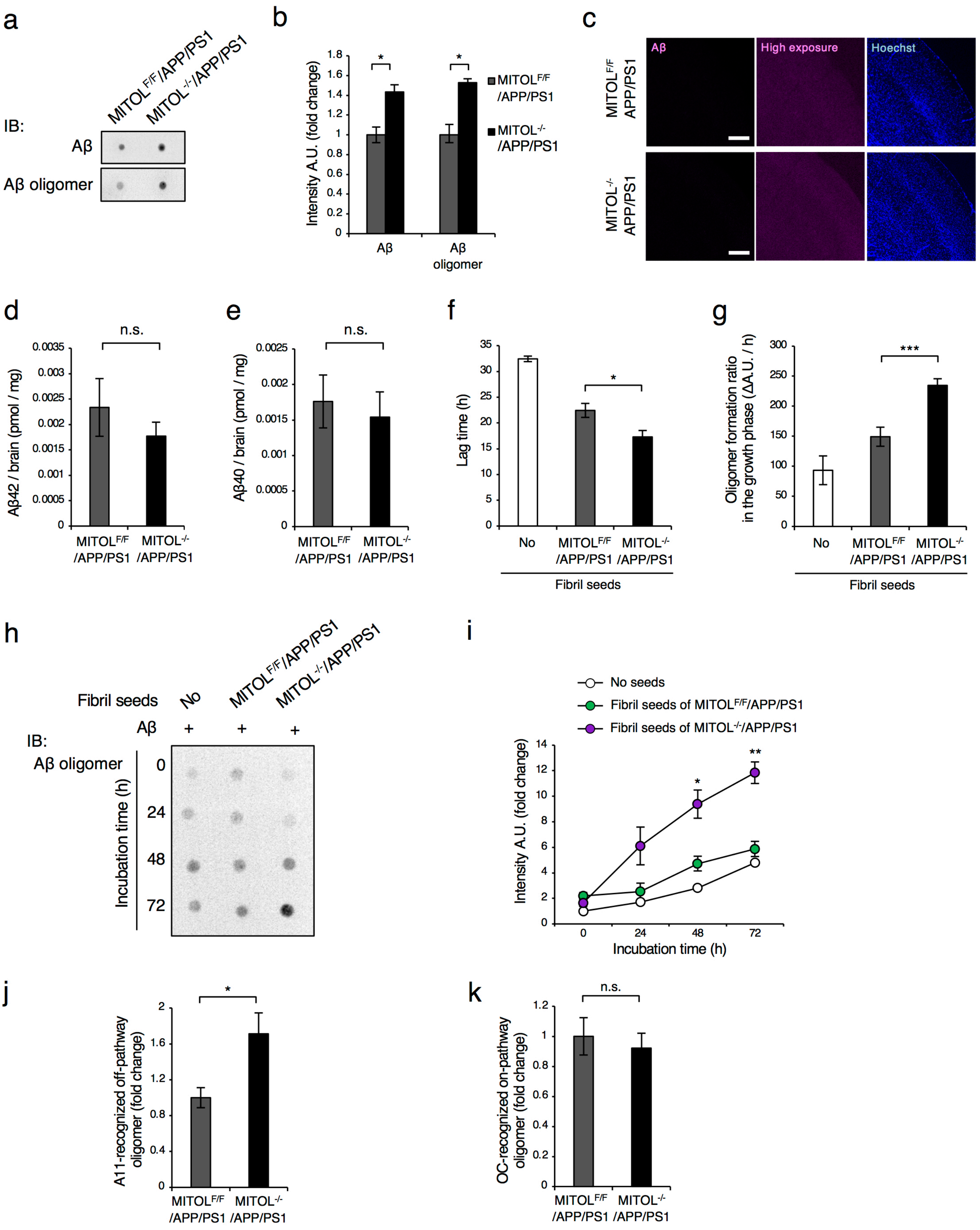

Supplementary Fig. 4. Characterization of Aβ oligomers in MITOL-deleted APP/PS1 mice.

a, **b** MITOL deletion induced the accumulation of soluble Aβ oligomers. TBS-soluble fraction was isolated from the cerebral cortex of indicated mice at 15 months of age to performed dot blot with anti-Aβ (6E10) or anti-Aβ oligomer specific (11A1) antibody (**a**), followed by the quantification (**b**). Error bars indicate ± SE (n=3-4). *p<0.05 (Student's t test). **c-e** MITOL did not regulate the accumulation of Aβ oligomers in the brain before Aβ plaque formation. Brain sections from indicated 3 months-old mice were immunostained with anti-Aβ antibody 6E10 and hoechst33258 (**c**). Scale bar represents 200 μm.

TBS-soluble fraction was isolated from the cerebral cortex of indicated mice at 3 months of ages to performed ELISA measurement of Aβ42 (**d**) and Aβ40 (**e**). Error bars indicate ± SE (n=5-6). n.s.: not significant (Student's t test). **f**, **g** Aβ fibrils from the MITOL-deleted APP/PS1 brain shortened the time up to the formation of new Aβ oligomers and facilitated the aggregation speed. Aβ fibrils in indicated 15-months old mice were isolated and subjected to the assay for monitoring the seeding activity of isolated Aβ fibrils as shown in Fig. 4d. The time up to the nucleation point and the ratio of fibril formation were calculated from Bis-ANS-monitored kinetic curves as lag-phase and elongation rate, respectively. Error bars indicate ± SE (n=4). $^{**}p<0.01$, $^{*}p<0.05$ (Student's t test). **h**, **i** Aβ fibrils from the MITOL-deleted APP/PS1 brain exerted a drastic seeding effect on the oligomerization of free Aβ40 monomers. Aβ fibrils including fibrillized plaques in indicated 15-months old mice were isolated and purified as described in method. The fibrils were co-incubated with seed-free Aβ40 monomers for indicated periods as described in method and the oligomerization was monitored dot blot with Aβ oligomer-specific antibody 11A1 (**f**). The intensity of Aβ oligomer is quantified (**g**). As control, TBS solution was used (No seeds). Error bars indicate ± SE (n=4). $^{**}p<0.01$, $^{*}p<0.05$ (Student's t test). **j**, **k** MITOL deletion led to dominant accumulation of off-pathway, out-of-register, Aβ oligomers. TBS-soluble fraction was isolated from the cerebral cortex of indicated mice at 15 months of age to performed sandwich ELISA measurement using anti-Aβ antibody 6E10 and either A11 or OC antibody to detect off-pathway oligomers or on-pathway oligomers, respectively. Error bars indicate ± SE (**j**: n=7-10, **k**: n=6-9). $^{*}p<0.05$, n.s.: not significant (Student's t test).

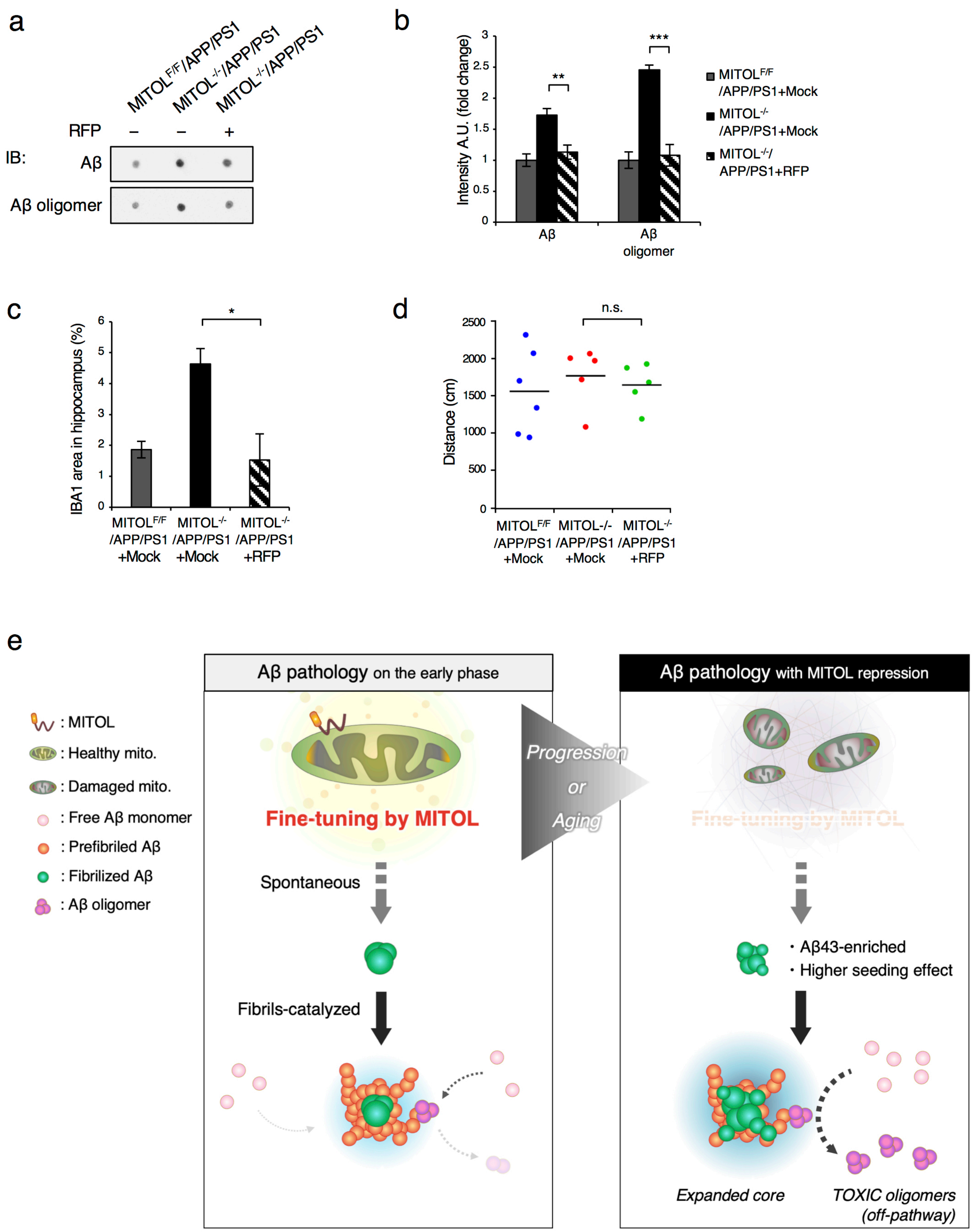

Supplementary Fig. 5. RFP affect only oligomeric Aβ.

a, **b** RFP treatment reduced soluble Aβ oligomers in MITOL-deleted APP/PS1 brain. RFP was treated as described in method. As control, the solvent for RFP was also treated (Mock). The cerebral cortex of 15 months-old mice is fractionated into TBS-soluble, followed by dot blot analysis with indicated antibodies. Error bars indicate ± SE (n=5-6). ***p<0.001, **p<0.01 (Student's t test). **c** Enhanced neuroinflammation in MITOL-deleted APP/PS1 mice was improved by RFP treatment. The percentage of IBA1-positive area in Fig. 4g was calculated using ImageJ. Error bars indicate ± SE (n=4). *p<0.05 (Student's t test). **d** The activity of mice was not changed by RFP injection. Open-field test was

conducted to quantify the activity of 15-month-old mice. Error bars indicate ± SE (n=5-6). n.s.: not significant (Student's t test). **e** A schematic illustration for the regulation of Aβ amyloidosis mediated by MITOL. Aβ forms plaques with fibrillized core, inducing the secondary-assembly of free Aβ monomers catalyzed by its seeding activity. MITOL mRNA expression is downregulated following the progression of Aβ pathology or aging. MITOL reduction or deletion in Aβ pathology contributes to perturbation of mitochondrial dynamics and faulty mitochondrial bioenergetics, leading to an alternative formation of Aβ fibrils with high ratio of Aβ43. The Aβ fibrils formed under the repression of MITOL resulted in the formation Aβ plaques with extended and large fibrillized core by an enhancing seeding activity. In addition, the Aβ43-enriched fibrils also modulated secondary-generation of soluble Aβ oligomers, characterized as dispersible and off-pathway, by the higher seeding effect for free Aβ monomers, thereby contributing severe Aβ pathology. mito.: mitochondria.

Supplementary Fig. 6.

Fig.1A

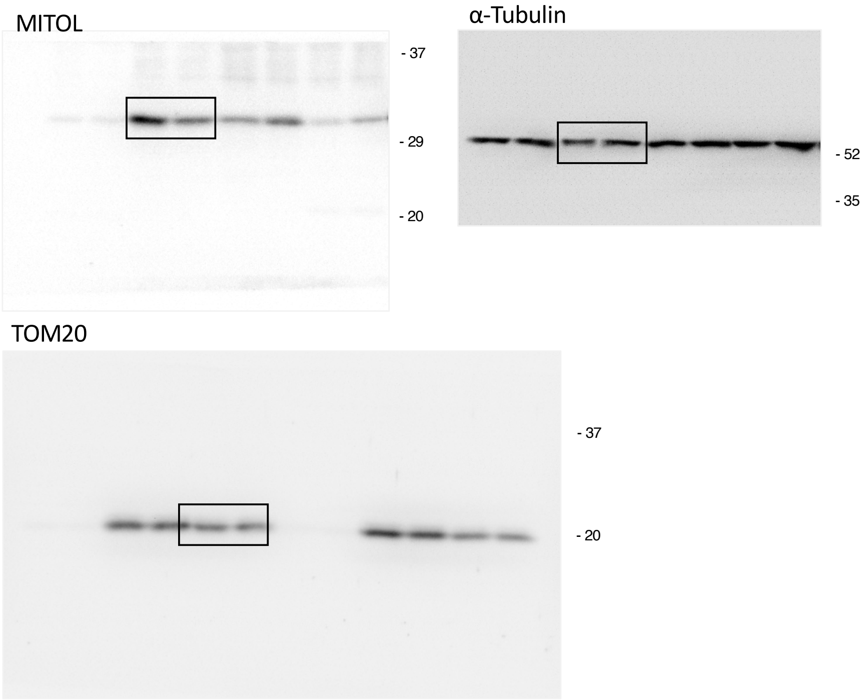

Fig.1C

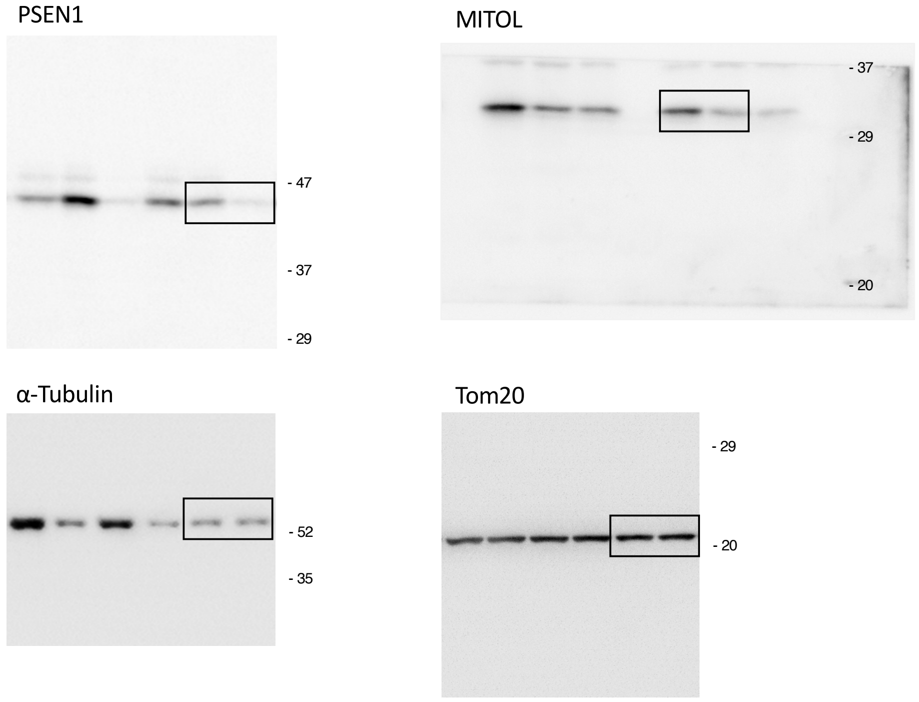

Supplementary Fig. 6. continued

Fig.2F

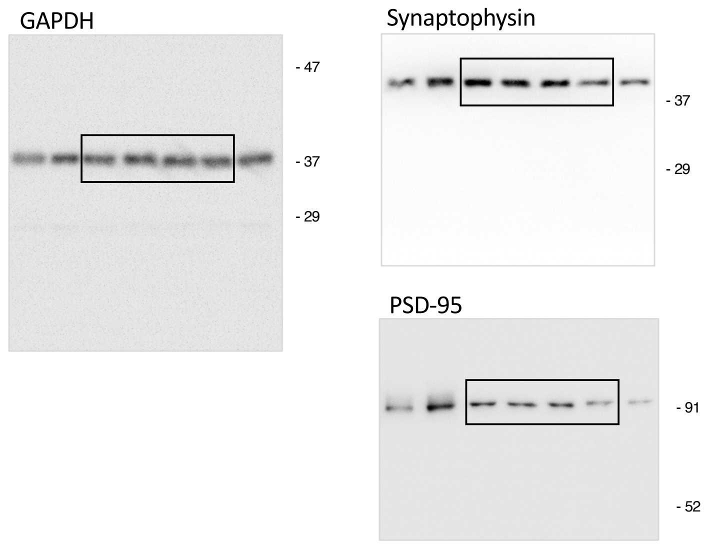

Fig.3B

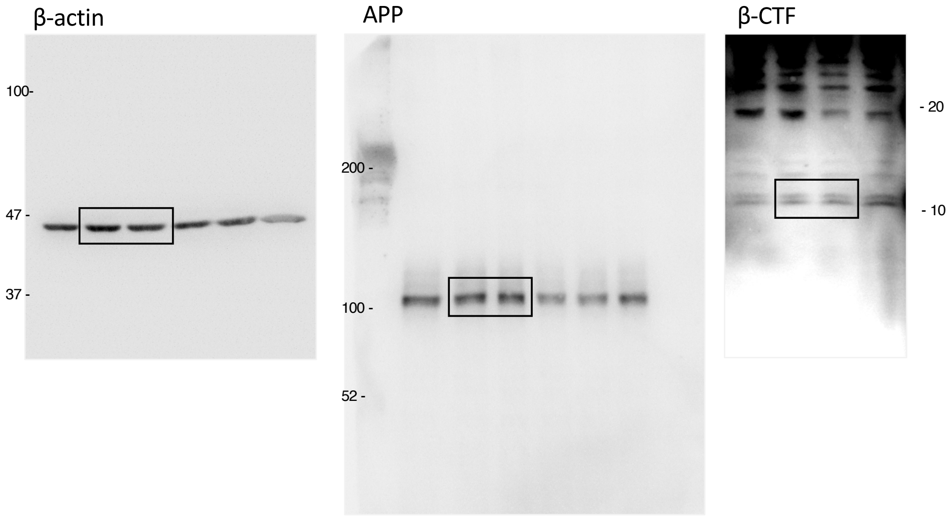

Fig.4G

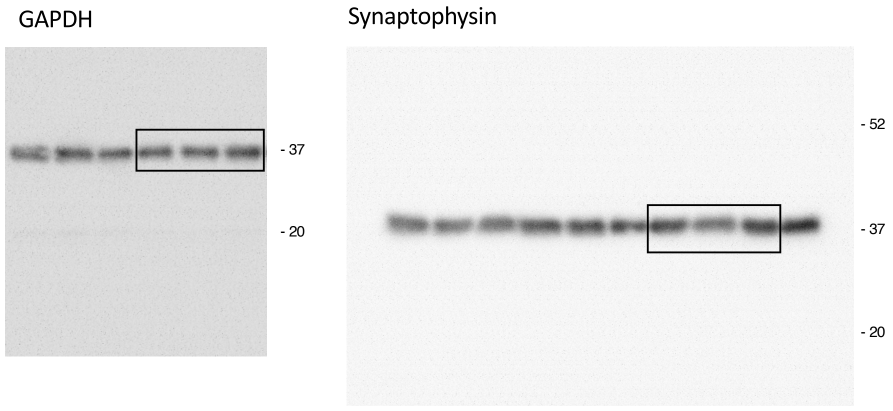

Supplement: Supplementary file 2 — Supplementary information [file 42003_2021_1720_MOESM2_ESM.pdf]
