# Supplementary material for: Mitochondrial ubiquitin ligase alleviates Alzheimer’s disease pathology via blocking the toxic amyloid-β oligomer generation
Source: Commun Biol. 2021 Feb 12;4:192. doi: 10.1038/s42003-021-01720-2 (PMC7881000; doi:10.1038/s42003-021-01720-2)
Supplement: Supplementary file 3 — Description of Supplementary Files [file 42003_2021_1720_MOESM3_ESM.pdf]

Description of Additional Supplementary Files

File Name: Supplementary Data 1

Description: Source data underlying the graphs in the main figures
